# Pharmacokinetics and tolerability of zibotentan (ZD4054) in subjects with hepatic or renal impairment: two open-label comparative studies

**DOI:** 10.1186/1472-6904-11-3

**Published:** 2011-03-17

**Authors:** Helen Tomkinson, John Kemp, Stuart Oliver, Helen Swaisland, Maria Taboada, Thomas Morris

**Affiliations:** 1 AstraZeneca, Alderley Park, Macclesfield, UK

## Abstract

**Background:**

Zibotentan (ZD4054) is a specific endothelin A (ET_A_) receptor antagonist being investigated for the treatment of prostate cancer. As zibotentan is eliminated by renal and metabolic routes, clearance may be reduced in patients with hepatic or renal impairment, leading to greater drug exposure.

**Methods:**

Open-label studies investigated the PK and tolerability of zibotentan in subjects with hepatic or renal impairment, compared with those with normal organ function. In the hepatic and renal studies, respectively, subjects were divided into categories using Child-Pugh classification or 24-hour urine creatinine clearance (mild, moderate, or severe impairment and normal function). Each subject received a single oral dose of zibotentan 10 mg and PK sampling was undertaken. Within the hepatic study, AUC and C_max _were expressed as the ratio of geometric means and 90% CI for each impairment group compared with the normal function group. The possibility that hepatic impairment had a clinically relevant effect on exposure was considered if the upper 90% CI for the ratio exceeded 2. In the renal study, AUC, C_max _and t_1/2 _were analyzed using linear regression fitting effects for creatinine clearance and age.

**Results:**

In the hepatic and renal studies respectively, 32 subjects (eight per group) and 48 subjects received treatment (n = 18 normal, n = 12 mild, n = 9 moderate, n = 9 severe). Zibotentan C_max _was not significantly affected by hepatic or renal impairment. Compared with the normal function group, zibotentan AUC was 40% (1.40; 90% CI 0.91-2.17), 45% (1.45; 90% CI 0.94-2.24) and 190% (2.90; 90% CI 1.88-4.49) higher in subjects with mild, moderate and severe hepatic impairment, respectively, and 66% (1.66; 90% CI 1.38-1.99), 89% (1.89; 90% CI 1.50-2.39) and 117% (2.17; 90% CI 1.64-2.86) higher in subjects with mild, moderate and severe renal impairment, respectively. In both studies mean t_1/2 _increased and zibotentan clearance decreased with the degree of impairment. Headache was the most common AE in all groups.

**Conclusions:**

Zibotentan absorption was unchanged, however, exposure was higher in subjects with hepatic or renal impairment due to slower clearance. This increased exposure did not result in differences in the range or severity of AEs observed.

**Trial Registration:**

ClinicalTrials.gov: NCT00672581 and AstraZeneca study number D4320C00016 (renal trial; conducted in Germany).

## Background

Prostate cancer is a leading cause of death in men in the Western world, accounting for an estimated 28% of new cancer cases in men in the US in 2010 [1]. Patients with advanced prostate cancer are initially treated with androgen deprivation therapy; however, disease progression will eventually occur in many men despite castrate serum androgen levels. This stage of disease is defined as castration-resistant prostate cancer (CRPC) for which treatment is currently limited to further hormonal manipulation or cytotoxic chemotherapy [2].

The endothelin (ET) axis has been implicated in several mechanisms that promote cancer progression. Endothelin-1 (ET-1) acting through the endothelin A (ET_A_) receptor is believed to promote tumour proliferation, angiogenesis, migration and invasion, as well as inhibiting apoptosis [3]. Conversely, activation of the ET_B _receptor by ET-1 promotes apoptosis and inhibits ET-1 production [4,5]. In prostate cancer, increased expression of the ET_A _receptor correlates significantly with increased tumour stage and aggressiveness whilst ET_B _receptor expression appears to be reduced or absent in CRPC [6]. Furthermore, activation of the ET_A _receptor by ET-1 is thought to be a key factor driving bone metastasis, which is a marked feature of CRPC [7,8]. In addition to its prominent role in CRPC, the ET axis has recently been implicated in a number of female malignancies including gynecological and breast cancers [9].

Zibotentan (ZD4054) is an oral specific ET_A _receptor antagonist in clinical development for the treatment of CRPC. A Phase II study of zibotentan monotherapy demonstrated a good tolerability profile and a promising overall survival signal in patients with metastatic CRPC who were pain free or mildly symptomatic for pain [10]. Zibotentan is being further assessed in a large Phase III clinical trial programme in this disease setting [11,12]. Preclinical investigations of zibotentan in other tumour types, including ovarian cancer, are ongoing [13,14].

Zibotentan exposure exhibited a dose-linear increase between 5 and 15 mg doses in Caucasian patients with CRPC. Following repeated dosing of zibotentan, there was minimal accumulation and no temporal change in the pharmacokinetics of zibotentan [15]. A pharmacokinetic (PK), metabolism and disposition study using [^14^C]-zibotentan has demonstrated that both renal excretion and metabolism are important clearance mechanisms for zibotentan [16]. The drug and its metabolites are predominantly eliminated in urine with ~58% of parent compound being eliminated by renal clearance. Metabolism of zibotentan is known to be mediated by the CYP3A4 isozyme [16,17]. When zibotentan was administered in combination with the potent inhibitor of CYP3A4, itraconazole, exposure evaluated by the area under the plasma concentration time curve from time zero to infinity (AUC) was increased by 28% [17]. Therefore, patients with hepatic or renal impairment may have reduced drug clearance which could potentially lead to a greater exposure to zibotentan than in patients with normal organ function.

Many patients with CRPC have acute renal failure due to obstruction of the urinary outflow tracts by the prostate tumour [18]. Furthermore, chemotherapy and some bisphosphonates, such as zoledronic acid, which are used widely in this disease setting, have also been associated with the development and progression of renal failure [19,20]. As zibotentan may be given to patients with CRPC prior to, or in conjunction with, chemotherapy and/or bisphosphonates it is important to establish whether the presence of hepatic or renal impairment has any impact on its exposure.

The aim of the two studies presented here was to determine whether hepatic or renal impairment (in subjects without CRPC) has any clinically relevant effect on exposure to zibotentan by assessment of PK, safety and tolerability parameters.

## Methods

### Study design and participants

#### Hepatic impairment study

This was an open-label, two-centre, single-dose, parallel-group study which assessed the effect of mild, moderate and severe hepatic impairment on the PK, safety and tolerability profile of zibotentan 10 mg. Subjects were divided into four groups (n = 8 per group [n ≥ 2 subjects of each sex per group]) using the Child-Pugh classification of hepatic impairment [21] based on scores for encephalopathy, ascites, serum bilirubin, serum albumin and prothrombin time (Table 1): normal hepatic function, matched to the hepatically impaired subjects with respect to age, gender and weight (control); mild hepatic impairment (Child-Pugh A); moderate hepatic impairment (Child-Pugh B); severe hepatic impairment (Child-Pugh C).

**Table 1:** Child-Pugh classification of hepatic impairment

	Points scored for observed findings		
	
	1 point	2 points	3 points
Encephalopathy grade*	Absent	1 or 2	3 or 4
Ascites	Absent	Slight	Moderate
Serum bilirubin (μmol/L)	<34.2	34.2-51.3	>51.3
Serum albumin (g/L)	>35	28-35	<28
Prothrombin time (INR)	<1.16	1.16-1.56	>1.56

**Classification**			

Child-Pugh grade	Child-Pugh A	Child-Pugh B	Child-Pugh C
Points required	5-6	7-9	10-15

INR, international normalized ratio. *Encephalopathy: Grade 0: normal consciousness, personality, neurological examination, electroencephalogram. Grade 1: restless, sleep disturbed, irritable/agitated, tremor, impaired handwriting, 5 cps waves. Grade 2: lethargic, time disorientated, inappropriate, asterixis, ataxia, slow triphasic waves. Grade 3: somnolent, stuporous, place disorientated, hyperactive reflexes, rigidity, slower waves. Grade 4: unrousable coma, no personality/behaviour, decerebrate, slow 2-3 cps delta activity.

Male and female subjects aged 18-75 years with a BMI of 18-34 kg/m^2 ^were included in the study. Subjects with normal hepatic function were required to be hepatitis B and C negative and have normal values for clinical laboratory tests and a normal medical history and examination. Females were to be surgically sterile or postmenopausal. Hepatically impaired subjects were required to have stable liver cirrhosis and hepatic impairment for at least 3 months prior to screening. Subjects were excluded if they had taken drugs with known significant cytochrome P450 (CYP) inducer/inhibitory effects within 30 days prior to zibotentan dosing; had abnormal resting vital signs of supine blood pressure >160 mmHg or <90 mmHg systolic or >95 mmHg or <50 mmHg diastolic or supine pulse ≥100 beats per minute (bpm) or ≤40 bpm; had a history or presence of gastrointestinal or renal disease or other condition known to interfere with the PK profile of drugs. Subjects were excluded from the control group if they had a history or presence of hepatic disease. Exclusion criteria from the hepatically impaired groups included: fluctuating or rapidly deteriorating hepatic function or presence of a hepatocellular carcinoma or an acute liver disease caused by drug toxicity or by an infection, significant renal dysfunction (creatinine clearance below 50 mL/min), severe portal hypertension (with exception of subjects in Child-Pugh C class) or surgical porto-systemic shunts, presence of severe hepatic encephalopathy, refractory ascites, or a platelet count below 40 × 10^9^/L and/or neutrophil count <1.5 × 10^9^/L and/or hemoglobin <90 g/L.

#### Renal impairment study

This was an open-label, single-centre, single-dose study which evaluated the effect of varying degrees of renal impairment on the PK, safety and tolerability profile of zibotentan 10 mg. Subjects were divided into four groups at screening (n = 12 per group [n ≥ 2 subjects of each sex per group, 50% of each group were to be >50 years]) using estimated creatinine clearance (CL_CR_) values (estimated using the Cockcroft-Gault equation) [22]; normal renal function (>80 mL/min); mild renal impairment (≥50 to ≤80 mL/min); moderate renal impairment (≥30 to <50 mL/min); severe renal impairment (<30 mL/min). Prior to analyzing the data, subjects were re-classified into their appropriate renal impairment groups based upon their measured creatinine clearance value determined using 24-hour urine collections on day -1 and serum creatinine levels obtained pre-dose on day 1.

Male and female subjects aged 25-75 years with a BMI of 18-32 kg/m^2 ^were included in the study. All subjects were required to be hepatitis B and C negative and all females were required to be surgically sterile or postmenopausal. Subjects with normal renal function were required to have normal values for clinical laboratory tests and a normal medical history and examination. Renally impaired subjects were to have had stable renal impairment for at least 2 months prior to zibotentan dosing. Subjects were excluded if they had taken drugs with known significant CYP inducer/inhibitory effects within 30 days prior to zibotentan dosing, had a history or presence of gastrointestinal or hepatic disease or other condition known to interfere with the PK of drugs. Subjects were excluded from the control group if they had a history or presence of renal disease, had abnormal resting vital signs of supine blood pressure >160 mmHg systolic or >100 mmHg diastolic or supine heart rate ≥90 bpm or ≤50 bpm. Exclusion criteria for renally impaired subjects included: renal transplant and end stage renal disease patients, use of drugs that affect creatinine clearance (such as cephalosporin antibiotics, ascorbic acid, trimethoprim, cimetidine and quinine) within 8 days of dosing and abnormal resting vital signs of supine blood pressure >180 mmHg or <110 mmHg systolic or >110 mmHg or <65 mmHg diastolic or supine heart rate ≥90 bpm or ≤50 bpm.

In both studies, all subjects received a single oral dose of zibotentan 10 mg and remained resident at the study unit from the night before the zibotentan dose was administered until 48 hours post dose.

The design of both trials followed the Food and Drug Administration (FDA) [23,24] and the European regulations [25,26] on the design and conduct of *in vivo *hepatic or renal impairment studies. These studies were performed in accordance with the Declaration of Helsinki [27], consistent with the ethical principles of the International Conference on Harmonization/Good Clinical Practice [28]. The hepatic study was approved by the Ethics Committee of the Institute for Clinical and Experimental Medicine and Faculty Thomayer Hospital, Prague, Czech Republic and the renal study was approved by the Bavarian Physicians Board, Ethics Committee, Munich, Germany. All subjects provided written informed consent prior to enrolment in the study and subsequent screening.

### Study objectives

The primary objective of these studies was to investigate the PK of a single oral dose of zibotentan 10 mg in subjects with hepatic or renal impairment compared with healthy subjects. The secondary objectives were to assess the safety and tolerability of a single oral dose of zibotentan 10 mg in these subjects.

### Procedures

Blood samples were collected for the determination of plasma concentrations of zibotentan pre dosing and at pre-defined intervals up to 96 and 120 hours, following receipt of a single oral dose of zibotentan 10 mg for subjects in the renal and hepatic impairment studies, respectively. Blood samples were centrifuged at 4°C for 10 minutes at 1500*g *to provide plasma. An additional blood sample was taken at 3 hours post dose for the determination of protein binding of zibotentan and was centrifuged at 37°C for 10 min at 1500*g *to provide plasma. Plasma samples were transferred into Amicon Centrifree cartridges (30,000 molecular cut off; Millipore, Watford). The cartridges were centrifuged in a fixed angle rotor at 1000-2000*g *at 37°C for 30 minutes to produce plasma ultrafiltrate. The collection cup was removed and stored at -20°C. In the renal impairment study, urine samples were collected from 0-6, 6-12, 12-24, 24-36 and 36-48 hours post dosing for the determination of zibotentan concentrations. The volume of each urine collection was recorded.

Plasma, plasma ultrafiltrate and urine samples for zibotentan analysis were stored at -20°C and transported to York Bioanalytical Solutions Ltd (York, UK). Zibotentan plasma and plasma ultrafiltrate concentrations were determined as described previously [17]. An additional calibration curve ranging from 5 to 5000 ng/mL which used an internal standard concentration of 10,000 ng/mL in water (rather than the 100 ng/mL solution previously reported for the 0.5 to 500 ng/mL calibration range) was used for plasma samples in the renal study and for plasma ultrafiltrate samples. The performance of the assay was monitored during each run using quality control samples at concentrations of 1.5, 200, 400 and 800 ng/mL where sample dilution was required (for the 0.5 to 500 ng/mL range) and 15, 2000 and 4000 ng/mL (for the 5 to 5000 ng/mL range) spiked into control human plasma samples or into control human plasma ultrafiltrate samples for the ultrafiltrate analysis. These were prepared prior to commencement of the analysis of study samples and stored at -20°C until required. In the hepatic study the CV of the assay was ≤12% at all concentrations and accuracy was typically between 98 and 103%. In the renal study the CV of the assay was ≤9.3% and the accuracy was typically 96 and 103%. Following analysis of the plasma ultrafiltrate samples, the CV of the assay was ≤8.4% in the hepatic study and ≤7% in the renal study and accuracy ranged from 101 to 106% and 97.8 to 107% for the hepatic and renal studies, respectively. Zibotentan urine sample concentrations were determined by dilution followed by HPLC with mass spectrometric detection (HPLC-MS-MS). Urine samples were aliquoted (100 μL) into polypropylene tubes with acetonitrile (100 μL). Samples were vortex mixed and sonicated at 40°C for 30 minutes. A 25 μL portion of each sample was aliquoted into a 2 mL square well plate and internal standard (900 μL, 1400 ng/mL) was added to each sample, except appropriate blanks, to which mobile phase (900 μL) was added. The plate was vortex mixed and centrifuged (3 minutes, 2500 rpm, 20°C), prior to being submitted for HPLC-MS-MS analysis as previously described [17]. The CV of the assay was ≤7.2% and the accuracy typically ranged from 101 to 107%.

Zibotentan PK parameters determined included maximum plasma concentration (C_max_), time to C_max _(t_max_), area under the concentration-time curve from zero to infinity (AUC), area under the concentration-time curve from zero until the last measurable concentration (AUC_0-t_), terminal half-life (t_1/2_), total apparent plasma clearance (CL/F), apparent volume of distribution at steady state (V_ss_/F), ratio of unbound drug in plasma (Fu), free C_max_, free AUC, and unbound CL/F. Renal clearance (CL_R_) and the fraction of dose excreted unchanged (Fe) were evaluated in the renal study only. Non-compartmental methods were used for the evaluation of the plasma concentration-time data and C_max _and t_max _were determined by inspection of the concentration-time profiles. Where possible, the terminal elimination rate constant (λ_z_) was calculated by log-linear regression of the terminal portion of the concentration-time profiles, and t_1/2 _was calculated as Ln2/λ_z_. AUC_0-t _was determined using the linear trapezoidal rule, and where appropriate, the AUC_0-t _was extrapolated to infinity using λ to obtain AUC. CL/F was calculated from the ratio of dose/AUC and Vss/F was determined from the mean residence time (MRT) × CL/F. The percentage of free zibotentan was determined by comparison of the free and total zibotentan concentrations at 3 hours post dose; free C_max _and free AUC were calculated using C_max _or AUC x percentage free zibotentan, respectively, and unbound CL/F was determined by CL/F/percentage free zibotentan. The amount of zibotentan excreted in the urine was determined from the concentration of zibotentan in each collection and the volume of urine collected. CL_R _of zibotentan was calculated from the total amount of zibotentan excreted/plasma AUC and the Fe of zibotentan was calculated as the total drug excreted unchanged/dose. The methods for the PK parameter assessments and calculations reported in this study have been described previously [29]. AUC, free AUC, AUC_0-t_, C_max_, and free C_max _were presented as geometric mean (CV) for each hepatic or renal study group. CL/F, unbound CL/F, t_1/2_, Vss/F, CL_R_, Fe and Fu were presented as arithmetic mean (± standard error [SE]).

Safety and tolerability was evaluated by recording the incidence of adverse events (AEs) according to Medical Dictionary for Regulatory Activities (MedDRA) vocabulary and the Common Terminology Criteria for Adverse Events (CTCAE) Version 3, laboratory tests (hematology, urinalysis and clinical chemistry), physical examination, and measurement of vital signs.

### Statistical methods

In the hepatic impairment study AUC and C_max _were logarithmically transformed using natural logarithms (back-transformed results were reported). These parameters were analyzed using an analysis of variance model (ANOVA) with a factor fitting for hepatic impairment status (mild/moderate/severe or normal). Ratios of geometric means of each hepatically impaired group compared to the normal function group (mild/moderate/severe: control) and 90% confidence intervals (CIs) were reported. An effect of hepatic impairment was predefined to have occurred if the upper 90% CI for the ratio did not lie below 2. This was chosen as zibotentan 15 mg has previously been tolerated in patients with CRPC; however, zibotentan 22.5 mg was not tolerated, therefore doubling of the zibotentan dose was to be eliminated [30].

For the renal impairment study, statistical analysis of AUC, C_max _(using natural logarithm transformed data) and t_1/2 _(using untransformed data) was performed using linear regression fitting effects for creatinine clearance and age as explanatory variables. The slope parameter and corresponding SE were used to provide point estimates and 90% confidence intervals (CIs) for the ratio (or difference for t_1/2_) of zibotentan exposures in subjects with severe, moderate and mild renal impairment compared to subjects with normal renal function.

## Results

### Patient demographics

Thirty-seven subjects were enrolled in the hepatic impairment study, 32 of whom received zibotentan and completed the study. In the renal impairment study, 52 subjects were enrolled and 48 subjects received zibotentan and completed the study. Twenty-four hour urine collections could not be taken until subjects had given consent and were admitted to the investigational site, therefore estimated creatinine clearance values using the Cockcroft-Gault equation were used at screening to classify subjects with varying degrees of renal impairment, to achieve 12 subjects per group. Subjects were subsequently re-classified within the renal impairment categories according to their actual serum creatinine clearance values obtained on day 1, resulting in a disproportionate number of subjects within each category. In both studies, all subjects were Caucasian, cohorts were balanced with respect to age and there were more males than females (Table 2).

**Table 2:** Demographic and baseline characteristics for subjects in the hepatic and renal impairment studies

		Degree of hepatic impairment				Degree of renal impairment		
				
	Normal hepatic function (n = 8)	Mild (n = 8)	Moderate (n = 8)	Severe (n = 8)	Normal renal function (n = 18)	Mild (n = 12)	Moderate (n = 9)	Severe (n = 9)
Male, n (%)	5 (63)	6 (75)	5 (63)	5 (63)	13 (72)	9 (75)	7 (78)	7 (78)
Female, n (%)	3 (38)	2 (25)	3 (38)	3 (38)	5 (28)	3 (25)	2 (22)	2 (22)
Mean age, years (range)	58.4 (55-62)	56 (45-63)	59.3 (49-68)	52 (37-67)	60 (47-71)	58 (38-71)	60 (48-69)	57 (32-69)

### Pharmacokinetics

#### Hepatic impairment study

**Figure 1:**
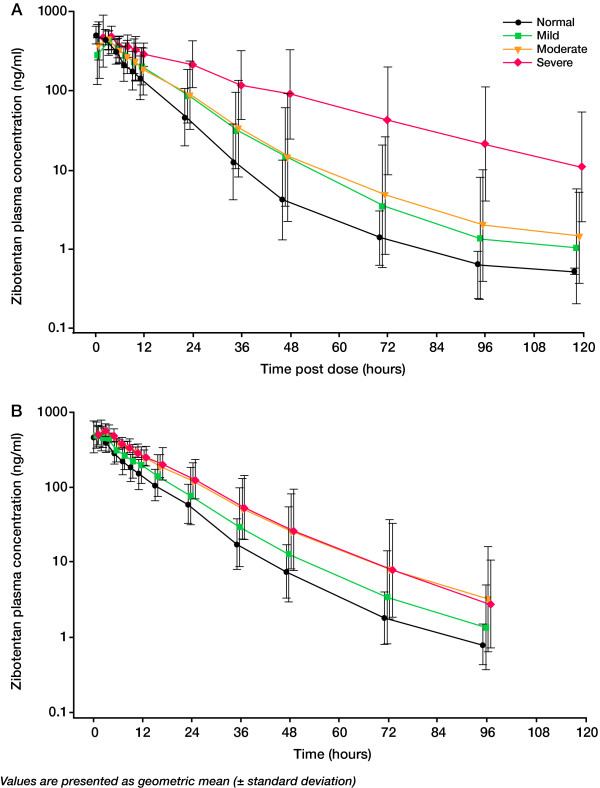
**Zibotentan plasma concentration-time curves**. Zibotentan plasma concentration-time curves for (a) subjects with normal hepatic function and varying degrees of hepatic impairment and (b) subjects with normal renal function and varying degrees of renal impairment.

**Figure 2:**
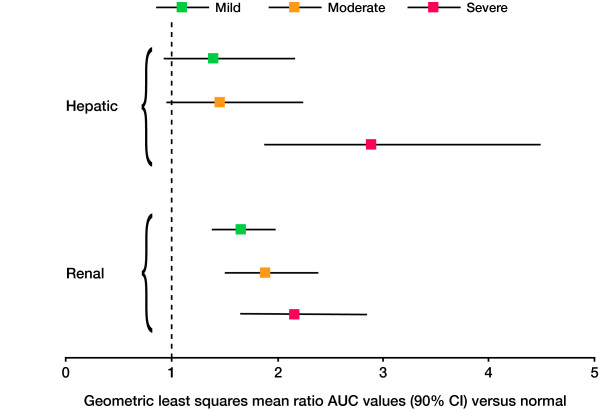
**Forest plot of the ratios of zibotentan exposure**. Forest plot of the ratios of zibotentan exposure (AUC) in subjects with varying degrees of renal impairment compared with subjects with normal renal function, and in subjects with varying degrees of hepatic impairment compared with subjects with normal hepatic function.

**Figure 3:**
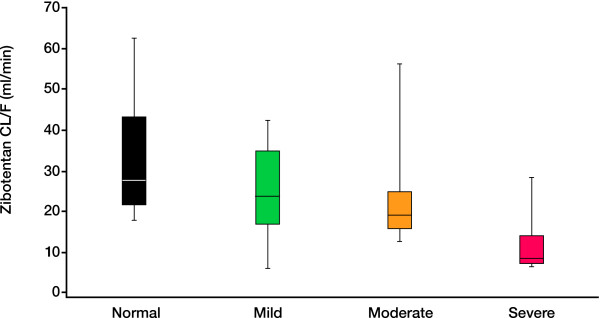
**Box plot of zibotentan clearance in subjects with normal function and varying degrees of hepatic impairment**.

The PK parameters and plasma concentrations of zibotentan 10 mg in subjects with varying degrees of hepatic impairment are presented in Table 3 and Figure 1a, respectively. The results of the statistical analysis are presented in Table 4 and Figure 2. Following a single oral dose of zibotentan 10 mg, C_max _was unchanged in subjects with mild, moderate and severe hepatic impairment compared with those with normal hepatic function (Table 4). Exposure in terms of AUC was significantly increased in subjects with hepatic impairment (Table 4; Figure 2). Zibotentan clearance (CL/F) was decreased in subjects with hepatic impairment with the magnitude of decrease being related to the degree of hepatic impairment (Figure 3). There was no statistical analysis of t_1/2 _values but the data demonstrated an increase in t_1/2 _in subjects with hepatic impairment compared with subjects with normal hepatic function (Table 3). The magnitude of the increase in this parameter was related to the degree of hepatic impairment. There was little difference in plasma protein binding between subjects with normal and impaired hepatic function, thus changes in free C_max_, free AUC and unbound CL/F across the groups were similar to changes in C_max_, AUC and CL/F (Table 3).

**Table 3:** Pharmacokinetic parameters of zibotentan in subjects with normal renal function and varying degrees of renal impairment, normal hepatic function and varying degrees of hepatic impairment

		Degree of hepatic impairment				Degree of renal impairment		
				
PK parameter	Normal hepatic function (n = 8)	Mild (n = 8)	Moderate (n = 8)	Severe (n = 8)	Normal renal function (n = 18)	Mild (n = 12)	Moderate (n = 9)	Severe (n = 9)
AUC_(0-t) _(ng·h/mL)*	5460 (46.2)	7560 (65.1)	7850 (50.3)	15100 (49.8)	5560 (36.9)	6910 (57.5)	9090 (35.2)	9640 (37.7)
AUC (ng·h/mL)*	5480 (46.0)	7680 (68.8)	7940 (50.7)	15900 (52.9)	5490 (39.0)^$^	6950 (58.3)	8710 (3.8)^£^	9750 (38.8)
C_max _(ng/mL)*	566 (25.6)	526 (22.3)	505 (23.0)	536 (30.2)	545 (22.7)	531 (28.8)	550 (9.9)	619 (20.6)
t_max _(h)^†^	2 (1-2)	2 (1-4)	2 (1-6)	2 (1-4)	1 (1-3)	1 (1-4)	2 (1-8)	1 (1-3)
t_1/2 _(h)^‡^	9.3 (3.6)	13.0 (9.4)	14.6 (6.5)	24.8 (10.9)	10.8 (2.7)^$^	11.3 (4.0)	13.5 (4.3)^£^	13.2 (4.7)
CL/F (mL/min)^‡^	33.2 (15.6)	25.0 (12.1)	23.6 (14.2)	11.9 (7.3)	32.7 (14.2)^$^	27.9 (18.9)	20.1 (6.5)^£^	18.2 (6.5)
V_ss_/F (L)^‡^	19.0 (6.1)	19.8 (3.1)	21.2 (7.2)	21.9 (7.1)	22.6 (7.0)^$^	20.8 (5.8)	19.3 (2.0)^£^	17.8 (3.4)
Fu (%)^‡^	22.5 (7.5)	23.4 (4.0)	20.2 (4.8)	29.2 (9.4)	22.8 (6.2)	25.4 (6.7)	26.6 (2.9)	27.9 (5.3)
CL_R _(mL/min)^‡^	-	-	-	-	17.4 (13.9)^$^	10.3 (16.8)	3.2 (5.6)^£^	2.3 (2.7)^£^
Fe (%)^‡^	-	-	-	-	47.2 (18.0)	27.1 (19.5)	12.7 (16.9)	10.5 (9.0)^£^
Free C_max _(ng/mL)*	121 (58.7)	123 (21.9)	97.3 (32.4)	149.2 (52.6)	121 (32.3)	131 (22.9)	145 (13.4)	170 (18.2)
Free AUC (ng·h/mL)*	1170 (69.3)	1800 (54.7)	1460 (56.5)	4430 (40.0)	1230 (39.1)^$^	1720 (60.6)	2260 (34.5)^£^	2680 (38.9)
Unbound CL/F (mL/min)^‡^	167 (98.4)	103 (46.8)	129 (69.8)	40 (14.7)	146 (67.3)^$^	115 (83.8)	77.4 (24.1)^£^	65.8 (22.3)

*Geometric mean (coefficient of variation, %), ^†^median (range), ^‡^arithmetic mean (standard deviation), ^$^n = 16, ^£^n = 8. AUC_(0-t)_, area under the plasma concentration-time curve from 0 to the time of the last quantifiable concentration; AUC, area under the plasma concentration-time curve from 0 to infinity; CL/F, total apparent drug clearance; CL_R_, renal clearance; C_max_, maximum plasma concentration; Fe, fraction of dose excreted unchanged; Fu, ratio of unbound drug in plasma; t_max_, time to reach C_max_; t_1/2, _terminal half-life; Vss/F, volume of distribution at steady state.

**Table 4:** Ratios of pharmacokinetic parameters of zibotentan in subjects with varying degrees of renal impairment compared with subjects with normal renal function, and in subjects with varying degrees of hepatic impairment compared with subjects with normal hepatic function

	Degree of hepatic impairment*			**Degree of renal impairment**^**†**^		
	
PK parameter	Mild	Moderate	Severe	Mild	Moderate	Severe
C_max _ratio (90% CI)	0.93 (0.75-1.15)	0.89 (0.72-1.10)	0.95 (0.77-1.17)	1.07 (0.97-1.19)	1.09 (0.96-1.24)	1.12 (0.96-1.30)
AUC ratio (90% CI)	1.40 (0.91-2.17)	1.45 (0.94-2.24)	2.90 (1.88-4.49)	1.66 (1.38-1.99)	1.89 (1.50-2.39)	2.17 (1.64-2.86)
t_1/2 _difference, h (90% CI)	-	-	-	1.87 (0.06-3.68)**	2.37 (0.08-4.66)**	2.87 (0.1-5.64)**

*Point estimate of geometric mean ratio in relation to control; ^†^Point estimate of geometric least squares ratio in relation to control; **Least squares mean difference in relation to control; AUC, area under the plasma concentration-time curve from zero to infinity; C_max_, maximum plasma concentration; t_1/2_, terminal half-life; CI = confidence interval.

#### Renal impairment study

**Figure 4:**
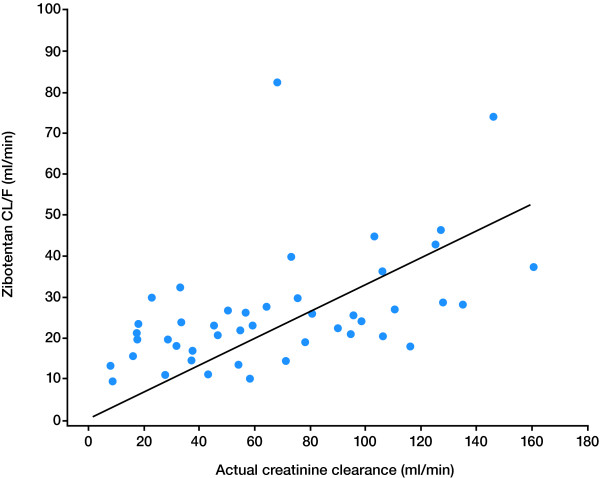
**Scatter plot of zibotentan clearance versus actual creatinine clearance in subjects with normal renal function and varying degrees of renal impairment**.

The PK parameters and plasma concentrations of zibotentan 10 mg in subjects with varying degrees of renal impairment are presented in Table 3 and Figure 1b, respectively. The results of the statistical analysis are presented in Table 4 and Figure 2. Following a single oral dose of zibotentan 10 mg, C_max _was unchanged in subjects with mild, moderate and severe renal impairment compared with subjects with normal renal function (Table 4). Exposure, in terms of AUC, was significantly increased in subjects with renal impairment and the magnitude of this increase was related to the degree of renal impairment; AUC was 66%, 89%, and 117% higher, respectively, in subjects with mild, moderate or severe renal impairment compared with subjects with normal renal function (Table 4; Figure 2). Zibotentan clearance (CL/F) decreased as the severity of renal impairment increased, with mean CL/F being 39% and 44% lower in the moderate and severe renal impairment groups, respectively, compared with subjects with normal renal function (Figure 4). Analysis of t_1/2 _indicated a difference with degree of renal impairment, with longer t_1/2 _values being observed as the severity of renal impairment increased (Table 3). There was little difference in plasma protein binding between subjects with normal and impaired renal function, thus changes in free C_max_, free AUC and unbound CL/F across the groups were similar to those observed for C_max_, AUC and CL/F (Table 3).

### Safety profile

Zibotentan was well tolerated in both studies and all AEs were CTCAE Grade 1 or 2. Headache (seven subjects [22%]) was the most common AE in the hepatic study which was reported in at least one subject in all groups, with the frequency increasing with the severity of hepatic impairment (Table 5). Vomiting was the second most common AE in the hepatic study, which was reported in two (6%) subjects. One subject with moderate hepatic impairment experienced a QT prolongation from 422 ms pre dose to 455 ms 4 hours post dose; however, this event was not considered to be related to zibotentan treatment and likely reflects normal variability in this parameter.

**Table 5:** AEs reported in >1 subject with normal hepatic function and varying degrees of hepatic impairment

		Degree of hepatic impairment		
		
Adverse event, n (%)	Normal hepatic function (n = 8)	Mild (n = 8)	Moderate (n = 8)	Severe (n = 8)
Any AE	1 (13)	1 (13)	3 (38)	4 (50)
Headache	1 (13)	1 (13)	2 (25)	3 (38)
Vomiting	1 (13)	0	0	1 (13)

Headache was also the most commonly reported AE in the renal impairment study; however, the incidence of headache did not appear to correlate with the severity of renal impairment (Table 6). Other AEs reported in more than two subjects included nasopharyngitis (n = 4), fatigue (n = 4), somnolence (n = 3) and nausea (n = 3).

**Table 6:** AEs reported in >1 subject with normal renal function and varying degrees of renal impairment

		Degree of renal impairment		
Adverse event, n (%)	Normal renal function (n = 18)	Mild (n = 12)	Moderate (n = 9)	Severe (n = 9)
Any AE	14 (78)	7 (58)	8 (89)	7 (78)
Headache	14 (78)	6 (50)	5 (56)	4 (44)
Nasopharyngitis	1 (6)	1 (8)	2 (22)	0
Fatigue	0	1 (8)	1 (11)	2 (22)
Somnolence	2 (11)	1 (8)	0	0
Nausea	1 (6)	1 (8)	0	1 (11)
Neck pain	0	2 (17)	0	0
Back pain	1 (6)	1 (8)	0	0
Dizziness	0	0	2 (22)	0
Dyspepsia	1 (6)	0	0	1 (11)

In both studies, AEs of headache were assessed by the investigator as being causally related to zibotentan treatment, and either resolved without medication or were managed with paracetamol. Minor reductions in systolic and diastolic blood pressure were noted following zibotentan dosing in most subjects in both studies; however, these changes were not associated with any symptoms and were not considered to be clinically relevant. There were no deaths, serious AEs, discontinuations due to AEs, or other significant AEs in either study.

## Discussion

A previous PK, metabolism and disposition study has indicated that zibotentan and its metabolites are predominately eliminated in urine. Between 71 and 94% of dosed drug is eliminated in the urine with 58% of an administered dose renally cleared as parent compound [16]. *In vitro *investigations have demonstrated that CYP3A4 is responsible for the metabolism of zibotentan [17]. Furthermore, when zibotentan was administered in combination with itraconazole, a potent inhibitor of CYP3A4, AUC increased by 28% [17]. Consequently, patients with hepatic or renal impairment may have reduced clearance of zibotentan, which could potentially lead to a greater exposure to zibotentan. A significant proportion of patients with CRPC are likely to have varying degrees of renal failure due to obstruction of the urinary outflow tracts by the tumour [18] and as a consequence of previous chemotherapy treatment regimens [19]. These observations support an assessment of the effects of hepatic or renal impairment on the PK of zibotentan.

The PK parameters of zibotentan in normal healthy subjects were similar between the two studies and were consistent with the findings of previous PK studies [17]. In subjects with mild, moderate or severe hepatic or renal impairment, there was no significant difference in the C_max _of zibotentan following a single oral dose of zibotentan 10 mg compared with those subjects with normal organ function, indicating that absorption of the drug was unchanged. Hepatic or renal impairment did, however, significantly increase zibotentan exposure (AUC), as a consequence of slower clearance of zibotentan. Furthermore, exposure increased with degree of hepatic or renal impairment. Of note, in the hepatic impairment study, the PK profile of one subject in the mild impairment group was similar to the PK profile of subjects in the severe impairment group and therefore the data from this subject will have influenced the mean PK values for the mild group and contributed to the wide variability observed (Figure 2). Indeed, when this subject was removed from the analysis, the upper confidence limit of the AUC treatment ratio fell below the predefined limit of 2. In both studies there was an increase in the elimination half-life of zibotentan as the degree of hepatic or renal impairment increased, although this was more evident in subjects with hepatic impairment. Hepatic and renal dysfunction has been shown to cause changes in plasma protein binding, therefore the fraction of unbound zibotentan was calculated at 3 hours post dose to determine free C_max_, free AUC and unbound CL/F. Little change was documented in protein binding across the groups in either study, and consequently changes in free C_max_, free AUC and unbound CL/F across the groups were similar to changes in C_max_, AUC and CL/F.

Data from the hepatic study have demonstrated that although mild and moderate impairment had only a small impact on the average PK profile of zibotentan, the impact of severe hepatic impairment was much greater. Total plasma clearance of zibotentan in individuals with severe hepatic impairment was 64% lower than that in individuals with normal function (Figure 3), resulting in an approximate 190% increase in exposure to zibotentan. Across the three hepatically impaired groups there was a large amount of variability. Although the average increase in exposure was 40 to 45% for the mild and moderately impaired groups, increases of more than 2 could not be ruled out. For the severely impaired group, increases of 4.5 fold could not be ruled out. Data from the renal study have shown that mild renal impairment had only a small impact on the PK profile of zibotentan with average exposure increasing 66% and the upper CI remaining below 2, whereas, in this case, the impact of moderate and severe renal impairment was progressively greater. Total plasma clearance of zibotentan in individuals with a moderate or severe degree of renal impairment was 39% and 44% lower, respectively, than in subjects with normal renal function, resulting in increases in zibotentan exposure of 89% and 117%, respectively.

In a Phase II study of zibotentan in patients with metastatic CRPC and bone metastases, zibotentan 15 mg was well tolerated, with headache being the most commonly reported AE [31]. Patients with mild renal impairment who receive zibotentan 10 mg may have exposures equivalent to those in patients receiving zibotentan 15 mg in the Phase II study, and therefore zibotentan is likely to be well tolerated. In contrast, in a Phase I study of patients with metastatic CRPC, patients taking zibotentan 22.5 mg reported dose-limiting toxicities of Grade 3 peripheral edema and intraventricular hemorrhage [30]. The most common AEs reported in this study were headache, peripheral edema, fatigue, nasal congestion, arthralgia and nausea. Groups of patients who get more than a doubling in mean drug plasma concentrations compared with normal patients could therefore be exposed to greater risks with zibotentan therapy. As such, caution and careful monitoring may be required if considering using zibotentan 10 mg/day in patients with hepatic insufficiency or moderate or severe renal insufficiency.

A single oral dose of zibotentan 10 mg was generally well tolerated in subjects with normal renal and hepatic function, and in those subjects with mild, moderate or severe hepatic or renal impairment. The most commonly reported AE in both studies was headache, which is consistent with reports from previous studies of zibotentan and other endothelin receptor antagonists [31,32]. The occurrence of headache increased with the degree of hepatic impairment, but not with the degree of renal impairment, where the incidence of headache was highest in subjects with normal renal function. In both studies, headache was reported to resolve without medication or was managed with paracetamol. Overall, there was an increase in the total number of AEs reported as the severity of hepatic impairment increased; 13% and 50% of subjects experienced AEs in the normal and severe hepatic impairment groups, respectively. In contrast, in the renal impairment study, the number of AEs reported was similar across all groups. This finding suggests that the increased exposure to zibotentan in subjects with moderate or severe renal impairment had little effect on the tolerability of zibotentan.

## Conclusions

Following administration of a single oral dose of zibotentan 10 mg to subjects with hepatic or renal impairment, the C_max _of zibotentan was unchanged, although zibotentan exposure (AUC) was higher in subjects with hepatic or renal impairment as a consequence of slower clearance of zibotentan. The magnitude of the increase in exposure was related to the degree of hepatic or renal impairment. Despite this increased exposure, there were no differences in the type or severity of AEs. Zibotentan 10 mg is currently undergoing further clinical investigation in patients with CRPC in a large Phase III clinical programme [11].

## Competing interests

This study was sponsored by AstraZeneca. Helen Tomkinson, John Kemp, Stuart Oliver, Helen Swaisland, Maria Taboada and Thomas Morris are all employees of AstraZeneca.

## Authors' contributions

HT performed the PK analysis of the renal study and drafted the manuscript. JK performed the PK analysis of the hepatic study. SO was the study physician in the renal study. MT performed the statistical analyses of both studies. TM and HS participated in the design and concept of the studies. All authors contributed to and approved the final manuscript.

## Pre-publication history

The pre-publication history for this paper can be accessed here:

http://www.biomedcentral.com/1472-6904/11/3/prepub
